# A new mathematical model for evaluating surface changes in the mid-abdominal sagittal plane after two-level pedicle reduction osteotomy in patients with ankylosing spondylitis

**DOI:** 10.1186/s12893-023-02285-z

**Published:** 2024-01-28

**Authors:** Wen Yin, Guohui Zheng, Wei Zhang, Yunlei Zhai, Haijiang Li, Lele Sun, Kangkang Wang, Jishi Jiang, Zikai Hua, Xilong Cui, Haiyang Yu

**Affiliations:** 1 Department of Orthopedics, Affiliated Fuyang People’s Hospital of Anhui Medical University, 501 Sanqing Road, Fuyang, 236000 Anhui China; 2 Spinal Deformity Clinical Medicine and Research Center of Anhui Province, 501 Sanqing Road, Fuyang, 236000 Anhui China; 3 School of Mechatronics Engineering and Automation, Shanghai University, 333 Nanchen Road, Shanghai, 200072 China

**Keywords:** Ankylosing spondylitis, Kyphotic deformity, Two-level pedicle subtraction osteotomy, Abdominal changes, mathematical model

## Abstract

**Background:**

The purpose of this study was to create a mathematical model to precalculate the acreage change in the abdominal median sagittal plane (ac-AMSP) of patients with ankylosing spondylitis (AS) for whom two-level pedicle subtraction osteotomy (PSO) was planned.

**Methods:**

A single-centre retrospective review of prospectively collected data was conducted among 11 adults with AS. Acreage of the abdominal median sagittal plane (a-AMSP) was performed. The distances and angles between the osteotomy apexes, anterosuperior edge of T12, xiphoid process, superior edge of the pubis, and anterosuperior corner of the sacrum were measured on preoperative thoracolumbar computed tomography. A mathematical model was created using basic trigonometric functions in accordance with the abdominal parameters. Planned osteotomized vertebra angles (POVAs) were substituted into the mathematical model, and the predictive ac-AMSP (P-AC) was obtained. A paired sample t test was performed to determine the differences between the P-AC and actual ac-AMSP (A-AC) and between the predictive acreage change rate (P-CR) and actual acreage change rate (A-CR).

**Results:**

The mean age and GK were 44.4 ± 8.99 years and 102.9° ± 19.17°, respectively. No significant difference exists between A-CR and P-CR via mathematical modeling (p > 0.05). No statistically significant difference existed between POVA and actual osteotomized vertebra angles (AOVA) (p > 0.05). A statistically significant difference was observed between preoperative and postoperative measurements of LL, SVA, and GK variables (*p* < 0.001).

**Conclusions:**

The novel mathematical model was reliable in predicting the ac-AMSP in AS patients undergoing two-level PSO.

**Supplementary Information:**

The online version contains supplementary material available at 10.1186/s12893-023-02285-z.

## Background

Ankylosing spondylitis (AS) is a chronic systemic rheumatic disease that mainly affects the sacroiliac joints and axial skeleton and can lead to severe thoracolumbar kyphosis in the late stage [1]. Corrective spinal osteotomy can effectively restore sagittal balance and reduce pressure on the abdomen and thorax [2]. Pedicle subtraction osteotomy (PSO) is regarded as a powerful procedure for correcting AS kyphotic deformity [3]. Many researchers have reported on the positive clinical and radiological results and high patient satisfaction rates of PSO techniques for fixed sagittal deformity. In addition to skeletal changes after PSO for the treatment of advanced-stage AS sagittal deformity, systemic changes after surgical treatment of kyphosis, including digestive function [4], abdominal skin [5], aortic length [6, 7] and aorta position changes [8], have been reported to influence patients’ quality of life. Liu [9] confirmed that the acreage of the abdominal median sagittal plane (a-AMSP) changes substantially in patients with severe AS kyphosis by PSO. Ji [10] performed a retrospective study among 29 patients with AS kyphosis after surgery. The average longitudinal diameter of the abdominal cavity increased substantially by 8.9 cm, and the average volume of the abdominal cavity increased by 652 mL.

However, to the best of our knowledge, there are no studies on precalculating the acreage change in the a-AMSP. Therefore, the aim of this study was to create a mathematical model that precalculates the acreage change in the abdominal median sagittal plane (ac-AMSP) in AS patients with rigid kyphotic deformity scheduled for two-level PSO.

## Materials and methods

### Patients

The present study was retrospective, and ethical approval was obtained from the ethical committee of the Affiliated Fuyang People’s Hospital of Anhui Medical University, China (Ethics Approval NO. 2022–1) on January 17, 2022. Eleven participants (9 men and 2 women) who met our criteria and were admitted between August 2017 and December 2019 were included in this study.

The inclusion criteria included adult patients who were diagnosed with AS and underwent preoperative and postoperative three-dimensional computed tomography (3DCT) scans of the thoracolumbar, had full-length spinal radiographs taken while standing in a neutral unsupported position, had rigid kyphotic deformity (RKD), and were undergoing two-level PSO closed-wedge surgery at the thoracolumbar level.

Our exclusion criteria included a history of spinal surgery, obvious coronal imbalance, PSO at a single level or above the T12 vertebral level, and additional osteotomy performed during the operation.

### Surgical technique: PSO

To achieve substantial kyphosis correction, a two-level PSO procedure was performed in this study [3, 11]. The osteotomy sites are usually at the apex of the deformity that contributes most to the deformity [12]. Under general endotracheal anaesthesia, the patient was placed in a prone position on an innovative adjustable prone positioning frame [13] that allowed the kyphotic spine to be accommodated and the kyphosis to be corrected simultaneously after the osteotomies.

The incision was made in the mid-beeline. The posterior components of the spine were exposed subperiosteally up to the transverse processes. Pedicle screws were inserted into two to four adjacent vertebrae cranial and caudal to the planned osteotomy site. A unilateral temporary rod was placed across the osteotomy site, and then the facet joint, lamina and pedicle were resected and PSO was performed. An L-shaped impactor was applied to break the posterior wall collapsing into the vertebral body. The dura and nerve root were completely released, and then the temporary rod was slowly loosened. As Zhang et al. [13] described, the height of the most proximal support module was kept constant. The heights of the remaining modules targeted from the apical area to the caudal area were successively lowered to the proximal height. A precise bent rod was installed while keeping the bending point and the osteotomy gap at the same level. The osteotomy gap was completely closed with the cantilever beam technique. Osteotomy was then performed at the second site, and closure was performed similarly to the first site. Correction was achieved through the closure of the posterior osteotomy without anterior column lengthening. After correction and instrumentation of the fixed sagittal deformity, posterolateral fusion was performed.

### Computed tomography measurements

The data measured below were used to develop a mathematical model for predicting the ac-AMSP in AS patients with rigid kyphotic deformity scheduled for two-level PSO. In this study, the a-AMSP was obtained using a previous method presented by Liu [4, 9]; this method was used to quantify abdominal acreage subtended by the following peripheries: (1) a beeline from the xiphoid process to the anterosuperior edge of T12, (2) a beeline from the xiphoid process to the superior edge of the pubis, (3) a beeline from the superior edge of the pubis to the anterosuperior corner of the sacrum, (4) a beeline from the anterosuperior corner of the sacrum to the inferior PSO’s apex, (5) a beeline from the inferior PSO’s apex to the superior PSO’s apex, and (6) a beeline from the superior PSO’s apex to the anterosuperior edge of T12. On the median sagittal plane, the following distances were measured: between the xiphoid process (X) and the superior edge of the pubis (P), between the superior edge of the pubis (P) and the anterosuperior corner of the sacrum (S), between the anterosuperior corner of the sacrum (S) and the inferior PSO’s apex (A_i_), between the inferior PSO’s apex and the superior PSO’s apex (A_s_), between the superior PSO’s apex (A_s_) and the anterosuperior edge of T12 (T), and between the xiphoid process (X) and the anterosuperior edge of T12 (T).

The distances between point X and point A_s_, and between point P and point A_i_ were also measured. The angles between beeline PA_i_ and beeline A_i_A_s_, and between beeline A_i_A_s_ and beeline XA_s_ were called *beta (β)* and *gamma (γ)*, respectively. The POVA was recorded preoperatively, and the actual osteotomized vertebra angle (AOVA) was obtained from the superior to the inferior endplate of the osteotomized vertebra after the operation. All patients underwent thoracolumbar computed tomography one week after surgery. The abovementioned measurements on 3DCT were performed on a computer using the iMedical centricity PACS system.

### Cobb angle measurement

Global kyphosis (GK) was measured through digital radiography from the superior endplate of the maximal thoracic vertebra to the inferior endplate of the maximal lower end vertebra. All values, including GK, thoracic kyphosis (TK), lumbar lordosis (LL), sagittal vertical axis (SVA) and computed tomography measurements, were measured twice by two independent observers, and the average was calculated.

### Mathematical modelling

**Fig. 1:**
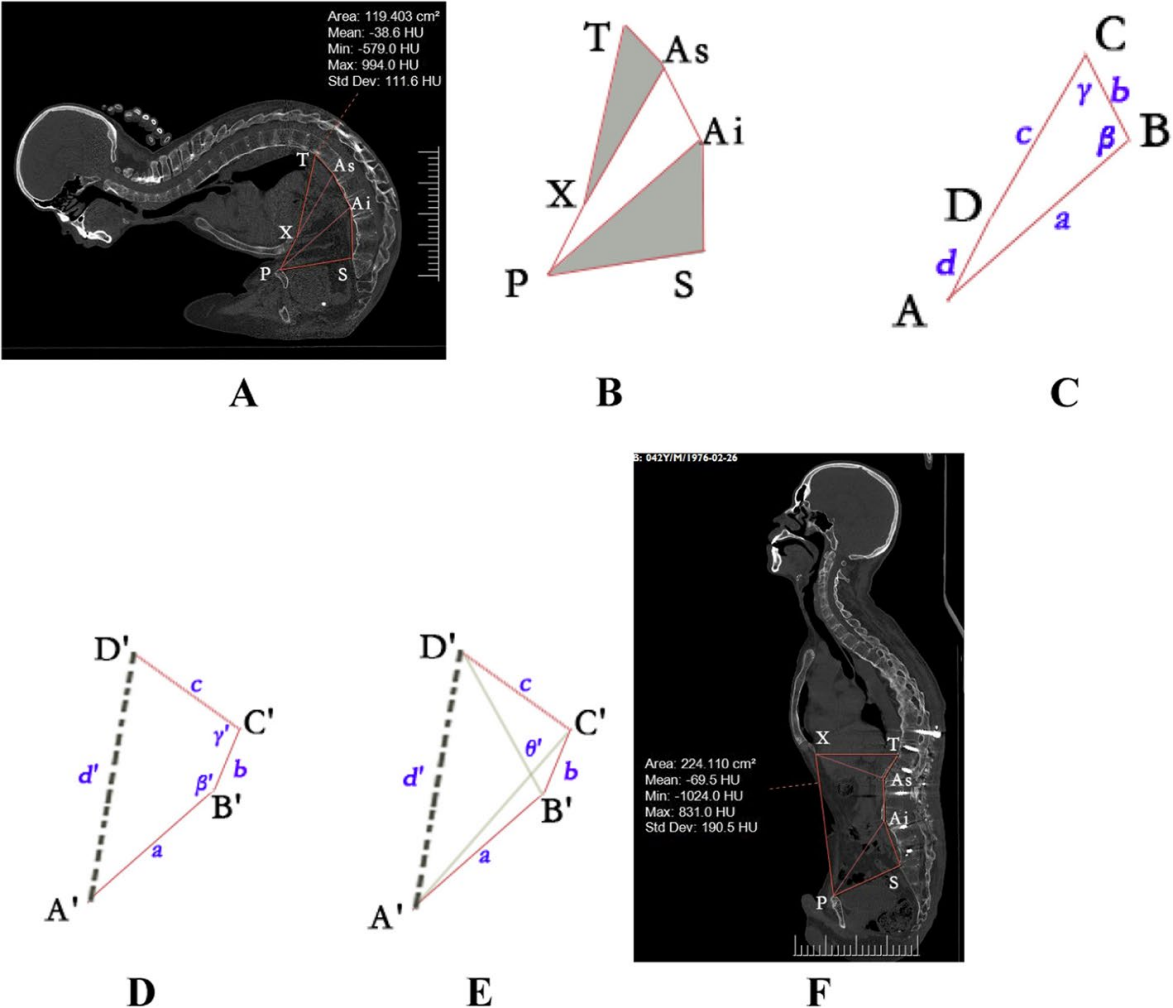
A 42-year-old male patient with ankylosing spondylitis with rigid kyphotic deformity (RKD) for whom two-level pedicle subtraction osteotomy (PSO) was planned. **A** Preoperative three-dimensional computed tomographic scans of the full spine showed that AS caused a stiff spine and abdominal compression. X is the point of the xiphoid process, P is the point of the superior edge of the pubis, S is the point of the anterosuperior corner of the sacrum, T is the point of the anterosuperior edge of T12, and A_i_ and A_s_ are the points of the inferior PSO’s apex and superior PSO’s apex, respectively. The acreage of the abdominal median sagittal plane (a-AMSP) can be obtained by measuring the preoperative hexagon acreage consisting of X, P, S, A_i_, A_s_, and T. **B** Under the condition of RKD with pedicle subtraction closing-wedge osteotomy, little changes are observed in the triangle XA_s_T and PSA_i_ area before and after the surgery. Meanwhile, the lengths of preoperative and postoperative XT, XA_s_, TA_s_, A_i_A_s_, PA_i_, PS, and SA_i_ are assumed to remain unchanged. Thus, acreage change in the abdominal median sagittal plane (ac-AMSP) is the rough equivalent of the preoperative and postoperative acreage changes in the quadrilateral consisting of X, P, A_i_, and A_s_. **C** To simplify the calculation, the lengths of PA_i_, A_i_A_s_, XA_s_, and XP were defined as a, b, c, and d, respectively. The preoperative points of P, Ai, As, and X were labeled as A, B, C and D, respectively. The angles between beeline a and beeline b, and between beeline b and beeline c were called beta(β) and gamma (γ), respectively. **D** After two separate osteotomies were performed at surgical osteotomy points B and C, the angle of β and γ increased to β'(β + α_1_) and γ'(γ + α_2_), respectively. α_1_ and α_2_ are the planed inferior and superior osteotomized vertebra angles, respectively. The postoperative points of P, A_i_, A_s_, and X were called A', B', C', and D', respectively. **E** θ' is the angle between A'C' and B'D'. The acreage of the quadrilateral consisting of A', B', C', and D' can be obtained as $S_{\left(A'B'C'D'\right)}=\frac{\sqrt{\left[a^2+b^2-2a\cdot b\cdot cos\;\left(\beta+\alpha 1\right)\right]\cdot\left[b^2+c^2-2b\cdot c\cdot cos\;\left(\gamma+\alpha 2\right)\right]-\left[b^2+a\cdot c\cdot cos\;\left(\beta+\gamma+\alpha 1+\alpha 2\right)-a\cdot b\cdot cos\;\left(\beta+\alpha 1\right)-b\cdot c\cdot cos\;\left(\gamma+\alpha 2\right)\right]^2}}2$, where $S\left(A'B'C'D'\right)$ is the acreage of the quadrilateral consisting of A', B', C', and D'. **F** Postoperative three-dimensional computed tomographic scans of the full spine showed two-level pedicle subtraction closing-wedge osteotomy surgery was performed at the first and third lumbar levels, and the inferior(α_1_) and superior(α_2_) osteotomized vertebra angles were 50° and 45°, respectively. The data of a, b, and c remain unchanged, but the numerical increase in d is evident. ac-AMSP is the rough equivalent of the preoperative and postoperative acreage changes in the quadrilateral consisting of X, P, A_i_, and A_s_

A mathematical model was created using basic trigonometric functions in accordance with the abdominal parameters. The measurement points were determined based on preoperative abdominal computed tomography, and the geometry was developed (Fig. 1).

The ac-AMSP could be obtained by subtracting the preoperative hexagon acreage consisting of X, P, S, A_i_, A_s_, and T from the postoperative hexagon acreage. Under the condition of rigid kyphotic deformity with pedicle subtraction closed-wedge osteotomy, little change occurred in the triangle XA_s_T and PSA_i_ area before and after the surgery. Moreover, the lengths of preoperative and postoperative XT, XA_s_, TA_s_, A_i_A_s_, PA_i_, PS, and SA_i_ were assumed to remain unchanged. Thus, the ac-AMSP was the rough equivalent of the preoperative and postoperative acreage changes in the quadrilateral consisting of X, P, A_i_, and A_s_. To simplify the calculation, the lengths of PA_i_, A_i_A_s_, XA_s_, and XP were defined as *a*,* b*,* c*, and* d*, respectively. The preoperative points of P, A_i_, A_s_, and X were called A, B, C, and D, respectively. The postoperative points of P, A_i_, A_s_, and X were called A', B', C', and D', respectively. The lengths of A'C' and B'D' were defined as *m*' *and n*', respectively. The angles between beeline A'B' and beeline B'C', beeline B'C' and beeline C'D', and beeline A'C' and beeline B'D' were called *β'*, *γ'*, and *θ', respectively*. The planed inferior and superior osteotomized vertebra angles were called $\alpha$_1_ and $\alpha$_2_, respectively, and the actual inferior and superior osteotomized vertebra angles were denoted as $\alpha$_1_' and $\alpha$_2_', respectively. The ac-AMSP during surgery was roughly equivalent to the change in quadrilateral ABCD.

The correction formula that changed the acreage of the abdominal median sagittal plane is as follows (Fig. 1):

$S\,(TXPSAiAs)$  *and *$S\,(T'X'P'S'Ai'As')$* are the preoperative and postoperative acreage of the hexagon acreage consisting of X, P, S, Ai, As, and T, respectively. *$S\,(ABCD)$  *and *$S\,(A'B'C'D')$* are the preoperative and postoperative acreage of the quadrilateral consisting of A, B, C, and D.*

$$ac\;-\;AMSP\;=S\,_{(T'X'P'Ai'As')}-S\,_{(TXPSAiAs)}\approx\;S\,_{\left(A'B'C'D'\right)}-\;S\,_{\left(ABCD\right)}$$

1. $S_{\left(ABCD\right)}$ and $S_{\left(TXPSAiAs\right)}$ can be measured directly through the iMedical centricity PACS system.
2. $S_{\left(A'B'C'D'\right)}=\frac{\sqrt{\left[a^2+b^2-2a\cdot b\cdot cos\;\left(\beta+\alpha1\right)\right]\cdot\left[b^2+c^2-2b\cdot c\cdot cos\;\left(\gamma+\alpha2\right)\right]-\left[b^2+a\cdot c\cdot cos\;\left(\beta+\gamma+\alpha1+\alpha2\right)-a\cdot b\cdot cos\;\left(\beta+\alpha 1\right)-b\cdot c\cdot cos\;\left(\gamma+\alpha2\right)\right]^2}}2$ (Please refer to Additional file 1 for the detailed imputation process of the formula) (1) and (2)

$$\text{ac}\;-\;\text{AMSP}\;=\frac{\sqrt{\left[a^2+b^2-2a\cdot b\cdot cos(\beta+\alpha 1)\right]\cdot\left[b^2+c^2-2b\cdot c\cdot cos(\gamma+\alpha 2)\right]-\left[b^2+a\cdot c\cdot cos(\beta+\gamma+\alpha 1+\alpha 2)-a\cdot b\cdot cos(\beta+\alpha 1)-b\cdot c\cdot cos(\gamma+\alpha 2)\right]^2}}2-S_{\left(ABCD\right)}$$

The change rate (CR) in the a-AMSP after the operation is given as

$$CR=\frac{S_{\left(T'X'P'S'Ai'As'\right)\,}-S_{\,\left(TXPSAiAs\right)}}{S_{(TXPSAiAs)}}\times100\%=\frac{\text{ac}-\text{AMSP}}{S_{(TXPSAiAs)}}\times100\%.$$

To facilitate the calculation and clinical promotion, the above mathematical model was created in Microsoft Excel software, through which we could easily obtain the required calculation results by inputting the preoperative measurement data (please refer to Additional file 2 for the mathematical model created in Microsoft Excel).

### Statistical analysis

Data analyses were performed with *SPSS 26.0* software (IBM Corp.). A paired sample t test was conducted to determine the differences between the lengths of preoperative and postoperative XT, XA_s_, TA_s_, A_i_A_s_, PA_i_, PS, and SA_i_. A paired sample t test was also performed to determine the ac-AMSP between the predicted and actual. Another paired sample t test was conducted to determine the differences between preoperative and postoperative TK, LL, SVA, GK, POVAs and AOVAs. Descriptive data are presented as the means and standard deviations. A *p* value of less than 0.05 was considered to be significant in all analyses.

## Results

The mean age of the participants was 44.4 ± 8.99 years. The mean GK was 102.9° ± 19.17°. The POVA was 38.4 ± 9.43, and the AOVA was 37.6 ± 10.54; no statistically significant difference was found between the POVA and AOVA (*p* = 0.083). Seven participants underwent PSO at L1 and L3, two participants at L2 and L4, and two participants at T12 and L3.

Table 1 lists the preoperative and postoperative parameters of the patients. No statistically significant difference was found between the preoperative and postoperative values of a, b, c, XT, TA_s_, SA_i_, PS lengths, and TK variables (*p* > 0.05). A statistically significant difference was observed between the preoperative and postoperative measurements of LL, SVA, GK, d, and the a-AMSP (*p* < 0.000, *p* < 0.000, *p* < 0.000, *p* < 0.000, and *p*: 0.010, respectively). No significant difference was observed between the A-AC and P-AC via mathematical modelling (*p*:0.301) or between the P-CR and A-CR (P:0.442) **(**Table 2).

**Table 1:** Preoperative and postoperative parameters of the patients

Parameters	Preoperative	Postoperative	df	95% CI	*p*
GK (°)	102.9° ± 19.17°	34.2° ± 19.35	10	55.6–81.7	< 0.000
LL (°)	-29.2 ± 26.50	39.9 ± 20.78	10	56.1–82.1	< 0.000
TK (°)	51.7 ± 21.85	49.1 ± 23.28	10	-1.6–6.8	0.204
SVA (cm)	350.0 ± 123.72	123.2 ± 57.57	10	156.2–297.3	< 0.000
a (cm)	17.5 ± 2.60	17.5 ± 2.50	10	-0.4–0.3	0.911
b (cm)	6.1 ± 1.16	6.4 ± 1.07	10	-0.6–0.1	0.124
c (cm)	13.5 ± 3.03	12.6 ± 2.55	10	-0.02–1.7	0.056
d (cm)	12.1 ± 7.01	26.7 ± 4.84	10	12.0–17.2	< 0.000
XT (cm)	14.0 ± 2.94	13.3 ± 2.90	10	-0.2–1.6	0.099
TA_s_ (cm)	3.8 ± 1.31	3.8 ± 1.43	10	-0.2–0.3	0.641
SA_i_ (cm)	8.2 ± 1.66	8.3 ± 1.81	10	-0.4–0.1	0.232
PS (cm)	11.4 ± 0.87	11.3 ± 0.82	10	-0.3–0.4	0.787
a-AMSP (cm^2^)	152.9 ± 42.01	208.1 ± 36.56	10	16.3–94.1	0.010*†*
*β* (°)	128.4 ± 24.09				
*γ* (°)	63.1 ± 17.42				

*GK* Global kyphosis, *LL* Lumbar lordosis, *TK* Thoracic kyphosis, *SVA* Sagittal vertical axis, a is the distance between the superior edge of the pubis and the inferior PSO’s apex, b is the distance between the inferior PSO’s apex and the superior PSO’s apex, *c* is the distance between the xiphoid process and the superior PSO’s apex, d is the distance between the xiphoid process and the superior edge of the pubis, *XT* is the distance between the xiphoid process and the anterosuperior edge of T12, TA_s_ is the distance between the anterosuperior edge of T12 and the superior PSO’s apex; SA_i_ is the distance between the anterosuperior corner of the sacrum and the inferior PSO’s apex; PS is the distance between the superior edge of the pubis and the anterosuperior corner of the sacrum; a-AMSP is the acreage of the abdominal median sagittal plane; β is the angle of the line between the connections of the superior edge of the pubis and the inferior PSO’s apex, and the inferior PSO’s apex and the superior PSO’s apex; γ is the angle of the line between the inferior PSO’s apex and the superior PSO’s apex, and the superior PSO’s apex and the xiphoid process; *df* degree of freedom, *95% CI* 95% confdence interval; †*p* < 0.05

**Table 2:** Parameters between the planed/predicted and actual OVA, AMSPAC, and CR

Parameters	Planed/predicted	Actual	df	95% CI	p
OVA (°)	38.4 ± 9.43	37.6 ± 10.54	21	-0.1–1.7	0.083
ac-AMSP(cm^2^)	53.5 ± 59.07	58.4 ± 56.03	10	-14.8–5.1	0.301
CR (%)	44.3 ± 38.00	45.8 ± 36.19	10	-0.1–0.03	0.442

*OVA* Osteotomized vertebra angle, *ac-AMSP* acreage change in the abdominal median sagittal planem, *CR* acreage change rate of the abdominal median sagittal plane, *df* degree of freedom, *95% CI* 95% confdence interval, statistical significance, *p* < 0.05

## Discussion

Kyphotic deformity secondary to AS can lead to sagittal imbalance, failure to look straight ahead, physical dysfunction, limited respiratory function, compression of the abdomen, and psychosocial problems [14]. Osteotomy is the most effective treatment method for advanced-stage AS. Surge ry during the advanced stage of AS often restores the sagittal balance by extension osteotomy, and it was initially described as open-wedge osteotomy, such as Smith‒Petersen osteotomy [15], but recently, it has been described as closed-wedge osteotomy, a protagonist of which is PSO [16]. Resection of the entire posterior column structure of the spine has become increasingly popular [16, 17]. Transpedicular closed-wedge osteotomy is safe to execute, likely because of the nature of the osteotomy, in which the two cancellous surfaces interact with quick healing and are initially stable because of the tension band nature of the posterior instrumentation and full anterior support [17]. To treat severe thoracolumbar kyphotic abnormalities in individuals with AS, single-stage, interrupted, two-level spinal osteotomy is an efficient and generally secure procedure [12]. In the present study, all patients had severe kyphotic deformity with a mean GK of 102.9° ± 19.17°, therefore two-level pedicle subtraction closed-wedge osteotomy was planned for all patients with AS and thoracolumbar kyphosis.

Most studies on AS are technically focused and describe the procedures, risks, and orthopaedic consequences of surgery on the spine. Favourable radiologic and clinical outcomes can also be realized after osteotomy. However, in the later stages of AS, substantial thoracic or thoracolumbar kyphotic deformity may cause the viscera to protrude from the abdomen by flexing the trunk, which reduces the volume of the abdominal cavity and weakens gastrointestinal motility. Liu et al. [9] reported that the preoperative and postoperative a-AMSP values were 172.106 ± 43.487 cm^2^ and 219.698 ± 30.449 cm^2^, respectively. In this study, the preoperative and postoperative a-AMSP were 152.9 ± 42.01 cm^2^ and 208.1 ± 36.56 cm^2^, resulting in an average increase of 58.4 ± 56.03 cm^2^. We believe that the reason for the difference in preoperative and postoperative a-AMSP values between this patient group and Liu's study is that all of our subjects had severe kyphotic deformity, excluding individuals with moderate deformity who were not candidates for a two-level osteotomy. The predicted ac-AMSP was 53.5 ± 59.07, which was not statistically different from the actual postoperative ac-AMSP. CR represents the ratio of the ac-AMSP to the preoperative a-AMSP. Variations among individuals meant that nobody else had the same preoperative a-AMSP. Using the CR rather than the ac-AMSP may be more appropriate to indicate the change degree of abdominal cavity volume. Patients in Liu's study that suffered from more severe kyphotic deformity had increased post-operative CR, and their body weight was heavier postoperatively [4, 9]. Additionally, the conditions of visceral extrusion by trunk flexion reduced the capacity of the abdominal cavity, and the osteotomy improved abdominal cavity pressure, which was followed by an improvement in digestive function. In the present study, the CR (P-CR: 44.3 ± 38.00%) could be predicted by mathematical model was not statistically different from the A-CR (45.8 ± 36.19%) after surgery. Predicting CR could be helpful when evaluating postoperative digestive function and providing patients with information about the potential risks along with the advantages of surgery.

Ji [10] discovered that while the transverse and anterior–posterior dimensions of the abdominal cavity remain unchanged after surgery, the longitudinal diameter increases dramatically by an average of 8.9 cm. It is suggested that alterations in sagittal morphology are the primary manner in which the volume of the abdominal cavity changes following spinal osteotomy. Therefore, while performing surgery on patients with advanced-stage AS, spine surgeons should be cognizant of these changes in abdominal cavity sagittal morphology [14]. In this study, the increase in abdominal longitudinal parameters was similarly observed. The distance of XP increased from 12.1 ± 7.01 cm to 26.7 ± 4.84 cm, which was obviously greater than the increase in the longitudinal diameter, More than that, even the aorta, the retroperitoneal organ of the abdominal cavity, was lengthened by an average of 2.2 cm with the increases of abdominal volume after osteotomy, causing a higher risk of significant vascular morbidity [10]. Although the prevalence of aortic rupture was minor, the results should be brought to our attention for the severity of this complication, with death caused by aortic rupture is hardly acceptable for a correction surgery. All measures should be taken to avoid this lethal complication [18]. Moreover, researchers have noted that the most common complications following surgery are those resulting from abdominal wall extension, even though they are not as serious as those arising from aortic rupture. Su et al. [5] assessed postoperative abdominal wall pain applying the Visual Analogue Score in 90 patients with AS thoracolumbar kyphosis. They discovered that the score for postoperative abdominal wall pain may reach 6.1 ± 2.7, and 53 patients had abdominal wall tension blisters measuring more than 5 mm. In a study on abdominal wall pain following thoracolumbar kyphosis deformity surgery in ankylosing spondylitis, Huang et al. [19] observed that 14 out of 15 patients in the control group had major postoperative symptoms related to abdominal wall pain. With the pubic symphysis at the lower end and the xiphoid process at the upper end, the abdominal wall is situated at the front edge of the abdomen. An acute increase in the XP value following orthopedic surgery corresponds with an increase in abdominal volume, which could be one of the causes of postoperative complications related to abdominal wall pain. In the present study, we found that the change of abdominal volume could be calculated using mathematical methods in advance among patients with AS with RKD for whom two-level PSO is planned. Predicting changes in abdominal volume appears to have potential as a perioperative method for assessing concerns.

The abdominal cavity was described as a large gap in the skeletal system between the lower edges of the thorax and the upper edge of the pelvis,which is closed by the muscles and their aponeuroses. The skeletal system, which is relatively fixed, provides attachment points for the soft tissue and muscles of the abdominal wall [20].The integrity of the abdominal wall is essential, not only in protecting the visceral structures, but also maintaining abdominal pressure [4, 9, 10, 20]. In patients with AS, the substantial narrowing of the gap between the thoracic and pelvic skeletons may result from the flexion and deformation of the thoracolumbar spine skeletal system in patients with AS. This was demonstrated in the current study by the significant raised XP value following spinal osteotomy. And this narrowing of the gap is accompanied by an increase in the abdominal pressure. In addition, variables that could contribute to decrease abdominal volume and subsequent rise in abdominal pressure might involve the concave folding of the abdominal wall and the abnormal rotation of the diaphragm. Thoracic activity tends to be considerably decreased in AS patients with severe rigid kyphosis. The diaphragm is a tissues that is located between thorax and abdominal cavity. Its activity might be restricted by limitations on thoracic mobility, which has the potential to decrease abdominal cavity's compensatory capacity. The diaphragm angle on median sagittal plane has changed significantly from -25.333º ± 4.283º to 18.222º ± 2.409º in patients with severe kyphosis in patients with ankylosing spondylitis who underwent PSO [9]. There was also significant improvement of both the minimum distance from the anterior abdominal wall to the spine and the a-AMSP in patients whose abdominal wall was folded into abdomen [9]. In addition to correcting abnormalities of the spinal bone structures, spinal osteotomy may also reduce the extent of diaphragm restriction, improve the concave and folded abdominal wall, increase abdominal volume, and alleviate abdominal pressure.

Corrective surgery for AS patients differs from that for other adult spine abnormalities since AS typically results in a stiff spine [16]. Given the few postoperative compensatory mechanisms for kyphosis caused by AS, especially for patients with RKD for whom two-level PSO is necessary, the trigonometric formula method can be used to calculate the osteotomized vertebra angle, osteotomy site, osteotomy midline, and global sagittal balance [21–23]. The centre of rotation is the anterior cortex of the vertebral body in pedicle subtraction closed-wedge osteotomy. The posterior and middle columns are shortened, but the anterior column is not lengthened, and anterior bone defects do not develop. Accordingly, when the posterior structure is closed with the anterior cortex of the vertebral body as the centre of rotation, the abdominal cavity in front of the vertebral body is extended, and the angle of abdominal extension is equivalent to the angle of osteotomy closure. Given that the morphology of the abdominal cavity is irregular, precise abdominal volume calculation is impossible, and no generalization can be performed. According to our research, abdominal volume variation is affected by a variety of factors, including osteotomized vertebra angle and demographic characteristics (e.g., PA_i_, A_i_A_s_, XA_s_, PA_s_, and XA_i_ distance), according to which a geometry can be built and a mathematical model can be established for predicting the postoperative change in abdominal volume.

Under the condition of having no change in the transverse and anterior–posterior diameters of the abdominal cavity after surgery [10] and substantially increasing the longitudinal diameter of the abdominal cavity and the acreage in the abdominal median sagittal plane [9], we consider that abdominal cavity volume can be assessed by the acreage change in the abdominal median sagittal plane. Additionally, patients with AS suffering from severe RKD have long-term contracture deformity of the trunk before surgery, and their chest and pelvis are often also stiff, accompanied by loss of rotation and compensatory functions. Thus, we assume that the lower edge of the thorax (XT beeline) and the upper edge of the pelvis (PS beeline) do not change after the operation, which has been confirmed by the results of the present study. In the present study, the distances of XT and PS were 14.0 ± 2.94 cm and 11.4 ± 0.87 cm, respectively, which were not statistically different from the postoperative, which were 13.3 ± 2.90 cm and 11.3 ± 0.82 cm, respectively. Although this assumption contradicts the previous views reported by Liu [4], we attribute the cause to the different patient populations that were selected. The correction formula for postoperative ac-AMSP is as follows:

$$\text{ac}\;-\;\text{AMSP}\;=\frac{\sqrt{\left[a^2+b^2-2a\cdot b\cdot cos(\beta+\alpha 1)\right]\cdot\left[b^2+c^2-2b\cdot c\cdot cos(\gamma+\alpha 2)\right]-\left[b^2+a\cdot c\cdot cos(\beta+\gamma+\alpha 1+\alpha 2)-a\cdot b\cdot cos(\beta+\alpha 1)-b\cdot c\cdot cos(\gamma+\alpha 2)\right]^2}}2-S_{\left(ABCD\right)}$$

The postoperative abdominal cavity volume change rate can be easily obtained as

$$CR=\frac{\text{ac}-\text{AMSP}}{S_{\left(TXPSAiAs\right)}}\times100\%.$$

The primary goal of closed-wedge osteotomy surgery for patients with AS is to ensure sagittal balance and horizontal view and reduce pressure on the abdomen and thorax [2]. The clinical and radiographic outcomes of closed-wedge osteotomy for AS kyphotic deformity have been reported by many authors. The decreased abdominal volume, visceral compression, and abnormal position of the organs resulting from the change in shape after kyphotic deformity could also be improved by spinal osteotomy, thereby improving the digestive function of AS patients [4]. Accurate prediction of postoperative alignment bears the potential to lower complication rates. The results of this study showed that the lengths of XP and the a-AMSP were significantly improved after surgery, and the change rate in abdominal cavity volume compressed by the kyphotic deformity could be predicted by the mathematical model. The mathematical modelling showed no significant differences between the A-CR and P-CR (*p* > 0.05). The unchanged lower edge of the thorax and upper edge of the pelvis were also proven by the results of the present study.

The purpose of this study was to introduce a method for predicting abdominal volume changes postoperatively. Due to the first mathematical model for calculating abdominal cavity volume changes, this study differs from other studies in the literature. However, our study has certain limitations and weaknesses. Retrospective studies can be biased by nature. Small sample size and gender disparity are limitations that cannot be ignored in this study. Since other causes of RKD were not included in this study, we do not know if the mathematical model can be applied to these patients. The mathematical model was adopted for two-level PSO only, and the changes in the lower edge of the thorax and upper edge of the pelvis were ignored. Moreover, as far as other spinal osteotomy technologies are concerned, we did not compute the effectiveness of this method. It is hoped that this formula will be tested for different spinal osteotomy technologies and different patient groups in the future.

## Conclusion

The proposed novel mathematical model is reliable in predicting the acreage change in the a-AMSP of patients with AS scheduled for two-level pedicle reduction osteotomy. The ability to predict CR may help with assessment of postoperative abdominal function and patient counseling regarding expectations and risks of surgery.

### Supplementary Information

**Additional file 1.**

**Additional file 2.**

## Data Availability

The patients’ data were collected in the Affiliated Fuyang People's Hospital of Anhui Medical University. The datasets used or analysed during the current study are available from the corresponding author upon reasonable request.

## Abbreviations

a-AMSP: Acreage of the abdominal median sagittal plane; ac-AMSP: Acreage change in the abdominal median sagittal plane
AS: Ankylosing spondylitis
PSO: Pedicle subtraction osteotomy
POVAs: Planed osteotomized vertebra angles
AOVAs: Actual osteotomized vertebra angles
P-AC: Predictive ac-AMSP
A-AC: Actual ac-AMSP
P-CR: Predictive acreage change rate
A-CR: Actual acreage change rate
CR: Change rate
GK: Global kyphosis
TK: Thoracic kyphosis
LL: Lumbar lordosis
SVA: Sagittal vertical axis
RKD: Rigid kyphotic deformity
