# Supplementary material for: A new mathematical model for evaluating surface changes in the mid-abdominal sagittal plane after two-level pedicle reduction osteotomy in patients with ankylosing spondylitis
Source: BMC Surg. 2024 Jan 28;24:38. doi: 10.1186/s12893-023-02285-z (PMC11370221; doi:10.1186/s12893-023-02285-z)
Supplement: Supplementary file 1 — Additional file 1. [file 12893_2023_2285_MOESM1_ESM.docx]

**Appendix 1: The detailed imputation process of the formula used for calculating the acreage change in the abdominal median sagittal plane of patients with** **ankylosing spondylitis after two-level pedicle subtraction osteotomy**

The specific imputation process of the formula $S_{（A'B'C'D'）}=\frac{\sqrt{\left[ a^{2}+b^{2}-2a\cdot b\cdot\cos\left( \beta+\alpha1 \right) \right]\cdot\left[ b^{2}+c^{2}-2b\cdot c\cdot\cos\left( \gamma+\alpha2 \right) \right]-\left[ b^{2}+a\cdot c\cdot\cos\left( \beta+\gamma+\alpha1+\alpha2 \right)-a\cdot b\cdot\cos\left( \beta+\alpha1 \right)-b\cdot c\cdot\cos\left( \gamma+\alpha2 \right) \right]^{2}}}{2}$ is as follows:


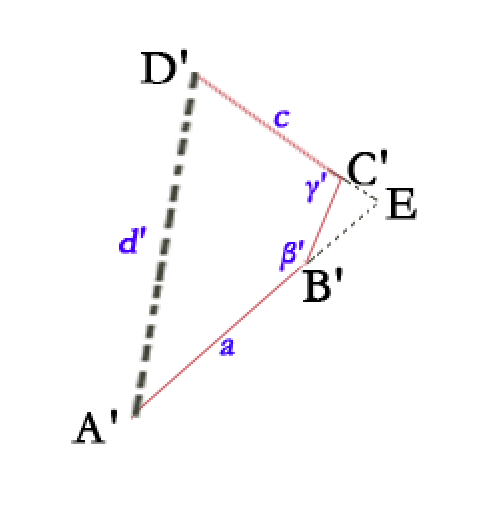
**
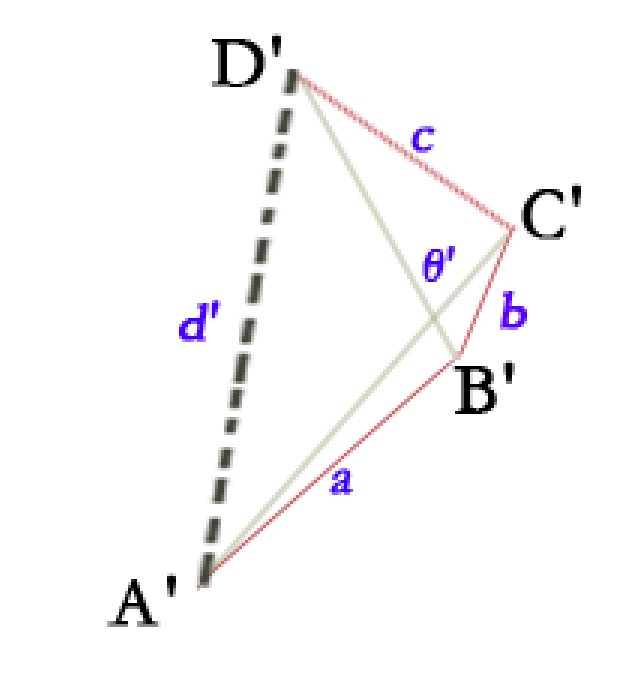
**

**A B**

Figure S1. The geometry of abdominal cavity after surgery. The postoperative points of the superior edge of the pubis(P), the inferior PSO’s apex(A_i_), the superior PSO’s apex(A_s_), and the xiphoid process(X) were called A', B', C', and D', respectively. The postoperative lengths of PA_i_, A_i_A_s_, and XA_s_ which remain unchanged before and after surgery were defined as a, b, and c, respectively. d' was the postoperative length of XP. The angles between beeline a and beeline b, and between beeline b and beeline c were called beta(β) and gamma (γ), respectively. α_1_ and α_2_ were the planed inferior and superior osteotomized vertebra angles, respectively. (A) After two separate osteotomies were performed, the angle of β and γ increased to β'(β+α_1_) and γ'(γ+α_2_), respectively. (B) θ' is the angle between A'C' and B'D'.

The extension lines of DC and AB intersect at point E, and ∠E is the angle between DA and AB.

∵(1)∠E+∠C'B'E+∠B'C'E=180°

(2) $\beta'$ +∠C'B'E=180°

(3) $\gamma'$+∠B'C'E=180°

∴(4)∠E=180°-∠C'B'E-∠B'C'E=$\beta'$+$\gamma'$-180°

According to the vector product formula and the law of trigonometric functions, we can obtain the following imputation process:

∵(5)$\overset{\to}{B'D'}=\overset{\to}{C'D'}-\overset{\to}{C'B'}$

(6)$\overset{\to}{A'C'}=\overset{\to}{B'C'}-\overset{\to}{B'A'}$

(7)∠E=180°-∠C'B'E-∠B'C'E=$\beta'$+$\gamma'$-180°

∴(8)$\overset{\to}{B'D'}\cdot\overset{\to}{A'C'}=\left( \overset{\to}{C'D'}-\overset{\to}{C'B'} \right)\cdot\left( \overset{\to}{B'C'}-\overset{\to}{B'A'} \right)=\overset{\to}{C'D'}\cdot\overset{\to}{B'C'}-\overset{\to}{C'D'}\cdot\overset{\to}{B'A'}-\overset{\to}{C'B'}\cdot\overset{\to}{B'C'}+\overset{\to}{C'B'}\cdot\overset{\to}{B'A'}=$b$\cdot$c$\cdot\cos(180-\gamma')-$a$\cdot$c$\cdot\cos(\beta'+\gamma'-180)+b^{2}-a\cdot b\cdot\cos\left( 180^{\circ}-\beta' \right)=b^{2}+a\cdot c\cdot\cos(\beta'+\gamma')-a\cdot b\cdot\cos\beta'-b\cdot c\cdot\cos\gamma'$

∵(9)$\left| \overset{\to}{A'C'} \right|=\sqrt{a^{2}+b^{2}-2a\cdot b\cdot\cos\beta'}$

(10)$\left| \overset{\to}{B'D'} \right|=\sqrt{b^{2}+c^{2}-2b\cdot c\cdot\cos\gamma'}$

(11)$\cos\theta'=\frac{\overset{\to}{B'D'}\cdot\overset{\to}{A'C'}}{\left| \overset{\to}{B'D'} \right|\cdot\left| \overset{\to}{A'C'} \right|}$

∴(12)$\cos\theta'=\frac{b^{2}+a\cdot c\cdot\cos\left( \beta'+\gamma' \right)-a\cdot b\cdot\cos\beta'-b\cdot c\cdot\cos\gamma'}{\sqrt{a^{2}+b^{2}-2a\cdot b\cdot\cos\beta'}\cdot\sqrt{b^{2}+c^{2}-2b\cdot c\cdot\cos\gamma'}}$

∵(13) $S_{（A'B'C'D'）}=\frac{1}{2}AC'\cdot BD'\cdot\sin\theta'$ and (9),(10),(12)

(14)$\sin\theta'=\sqrt{1-\cos^{2} \theta'}$

(15)$\beta'=\beta+\alpha1$

(16)$\gamma'=\gamma+\alpha2$

∴(17)$S_{（A'B'C'D'）}=\frac{\sqrt{\left[ a^{2}+b^{2}-2a\cdot b\cdot\cos\left( \beta+\alpha1 \right) \right]\cdot\left[ b^{2}+c^{2}-2b\cdot c\cdot\cos\left( \gamma+\alpha2 \right) \right]-\left[ b^{2}+a\cdot c\cdot\cos\left( \beta+\gamma+\alpha1+\alpha2 \right)-a\cdot b\cdot\cos\left( \beta+\alpha1 \right)-b\cdot c\cdot\cos\left( \gamma+\alpha2 \right) \right]^{2}}}{2}$
